# Simulating Upper Eyelid Ptosis During Neuromodulator Injections—An Exploratory Injection and Dissection Study

**DOI:** 10.1111/jocd.16631

**Published:** 2024-10-12

**Authors:** Ferdinando Paternostro, Wei‐Jin Hong, Guo‐Sheng Zhu, Jeremy B. Green, Milan Milisavljevic, Mikaela V. Cotofana, Michael Alfertshofer, S. Benoit Hendrickx, Sebastian Cotofana

**Affiliations:** ^1^ Department of Experimental and Clinical Medicine, Anatomy and Histology Section University of Florence Florence Italy; ^2^ Department of Plastic and Reconstructive Surgery Guangdong Second Provincial General Hospital Guangzhou China; ^3^ Skin Associates of South Florida and Skin Research Institute Coral Gables Florida USA; ^4^ Laboratory for Vascular Morphology Institute of Anatomy, Faculty of Medicine, University of Belgrade Belgrade Serbia; ^5^ Cotofana Anatomy Rochester Minnesota USA; ^6^ Department of Oral and Maxillofacial Surgery Ludwig‐Maximilians‐University Munich Munich Germany; ^7^ Department of Plastic and Reconstructive Surgery University Hospital Leuven Leuven Belgium; ^8^ Department of Dermatology Erasmus Medical Centre Rotterdam The Netherlands; ^9^ Centre for Cutaneous Research Blizard Institute, Queen Mary University of London London UK; ^10^ Department of Plastic Surgery Vanderbilt University Medical Center Nashville Tennessee USA

**Keywords:** adverse events, aesthetic medicine, eyelid ptosis, levator palpebrae superioris muscle, neuromodulator injections

## Abstract

**Background:**

Aesthetic neuromodulator injections of the upper face are frequently performed to temporarily block muscular actions of the periorbital muscles to ultimately reduce skin rhytids. However, the adverse event rate in the literature for toxin‐induced blepharoptosis ranges from 0.51% to 5.4%.

**Objective:**

To identify access pathways by which injected neuromodulator product can travel from extra‐ to intra‐orbital and therefore affect the levator palpebrae superioris muscle.

**Methods:**

Nine non‐embalmed human body donors were investigated in this study with a mean age at death of 72.8 (16.1) years. The 18 supraorbital regions were injected in 28 times (14 for supratrochlear and 14 for supraorbital) with 0.5 cc, whereas eight cases (four for supratrochlear and four supraorbital) were injected with 0.1 cc of colored product. Anatomic dissections were conducted to identify structures stained by the injected color.

**Results:**

The results of this injection‐ and dissection‐based study revealed that both the supratrochlear and the supraorbital neurovascular bundles are access pathways for injected neuromodulator products to reach the intra‐orbital space and affect the levator palpebrea superioris muscle. Out of 36 conducted injection passes, seven (19.44%) resulted in affection of the sole elevator of the eyelid of which 100% occurred only at an injection volume of 0.5 cc and not at 0.1 cc.

**Conclusion:**

Clinically, the results indicate that a low injection volume, a superficial injection for the supraorbital location, and angling the needle tip away from the supratrochlear foramen (toward the contralateral temple) when targeting the corrugator supercilii muscles, can increase the safety profile of an aesthetic toxin glabellar treatment.

## 1 Introduction

The number of conducted neuromodulator injections in the United States reached in 2023 9 480 949 procedures, which represents an increase of 9% when compared to 2022 [1]. This reflects on the acceptance of minimally invasive procedures and on its constant growth during the last decades. Since its first introduction into the aesthetic market in 1989 [2], neuromodulators have been shown to be effective when it comes to temporarily paralyzing facial muscles and thereby reducing facial rhytids [3, 4].

The injection locations where neuromodulators are administered correspond with the anatomic locations of facial muscles establishing a strong connection between facial muscle anatomy and effectiveness of the treatment. However, such relationship is depending not only on the 2D location of facial muscles but also on their 3D location, that is, the fascial planes [5, 6]. For example, the corrugator supercilii muscle (CSM) should be targeted medially deep to reach its muscle belly, whereas laterally the muscle should be injected superficially to best target its dermal insertion and its connection with the orbicularis oculi muscle (OOM) [7]. The OOM overlies the orbital septum which is thought to provide a fascial boundary between extra‐ and intra‐orbital structures. The supratrochlear and supraorbital neurovascular bundles, however, emerge from inside of the orbit and either pierce the orbital septum (when the vessels hook around the orbital rim) or bypass it via a foramen thereby creating a potential access pathway from extra‐ to intra‐orbital.

Despite being of temporary effectiveness, adverse events have been reported to be associated with the administration of neuromodulators. For upper face treatments, the most common toxin‐related adverse events are eyebrow ptosis and eyelid ptosis, also termed blepharoptosis [8, 9]. The frequency of the latter adverse event type is published in the scientific literature to range between 0.51% and 5.4% and has been associated with the affection of the levator palpebrae superioris (LPS) muscle [10, 11, 12]. Neuromodulator product reaching and being in contact with the LPS results in its temporary inability to contract and to lift the upper eyelid causing the patient visual discomfort for the duration of toxin activity.

A previous study by Nestor et al. [12] summarized precautionary measures that should help to mitigate the risk for causing eyelid ptosis which include the administration of product at least 1 cm cranial to the eyebrow cilia, applying digital pressure over the supraorbital rim, and pointing the needle away from the orbit, when targeting the glabella.

Despite the understanding that there is a connection between the injection of the neuromodulator and eyelid ptosis, the exact pathway by which the product reaches the LPS remains elusive. Therefore, the objective of this study was to conduct an anatomic exploratory study in which colored product is injected in the supratrochlear and supraorbital regions and traced into the orbit to identify the access pathway by which the product reaches the LPS.

## 2 Material and Methods

### 2.1 Study Design

This anatomic injection‐ and dissection‐based study was conducted to identify the spread of colored water within the supraorbital soft tissues. The period in which the study was carried out was from December 2023 to July 2024. A total of nine human non‐embalmed body donors were utilized to simulate supraorbital neuromodulator injections. The study was conducted at the ICLO Teaching and Research Center, Verona, Italy and at the Department of Plastic and Reconstructive Surgery, Guangdong Second Provincial General Hospital, Guangzhou, Guangdong Province, China.

Ethics approval was not deemed necessary because of the cadaveric nature of this study relying on the local laws in which the injections and dissections were conducted (Italy and China).

### 2.2 Injection Protocol

**FIGURE 1:**
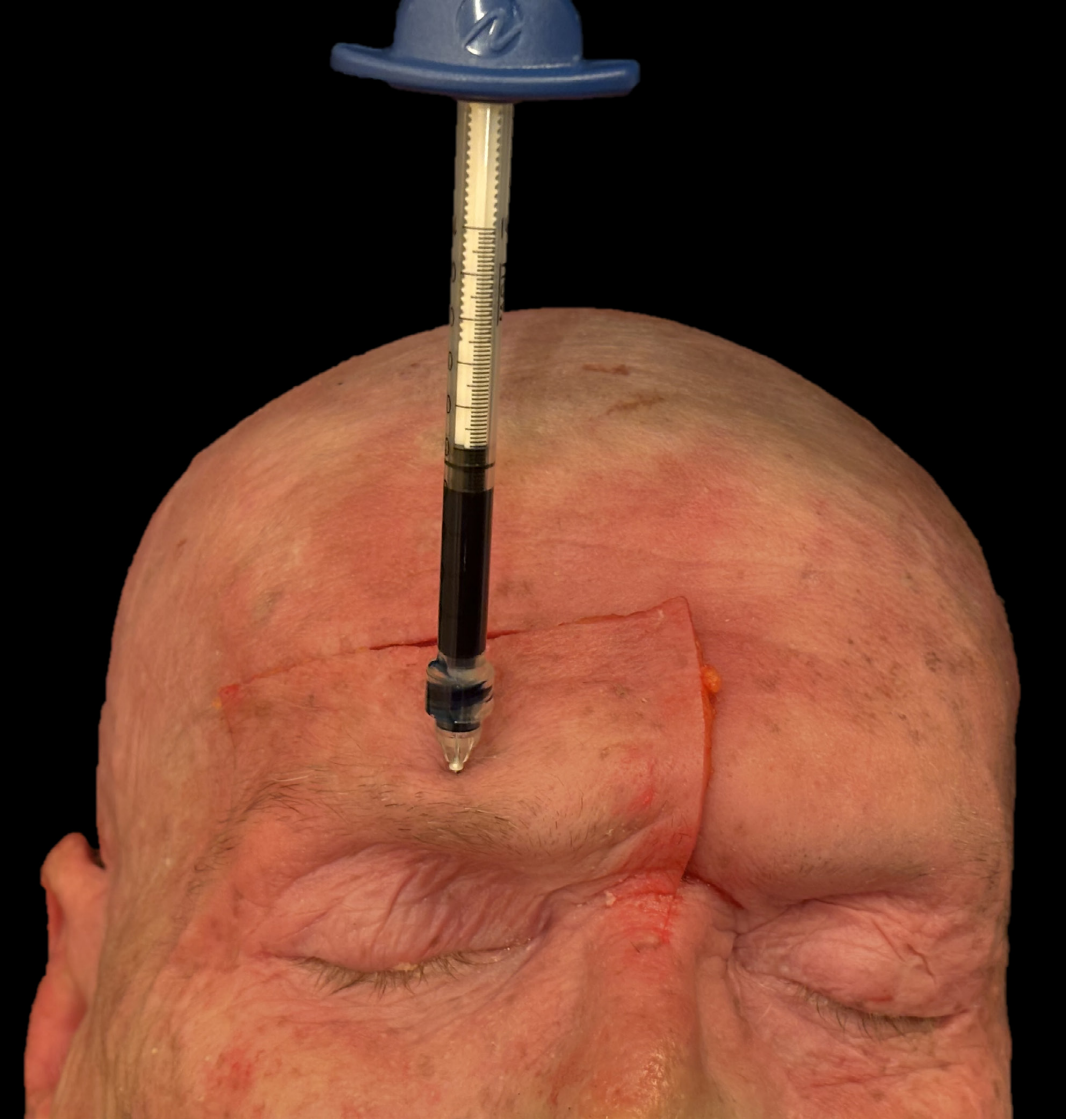
Demonstration of the injection technique for the supraorbital region.

**FIGURE 2:**
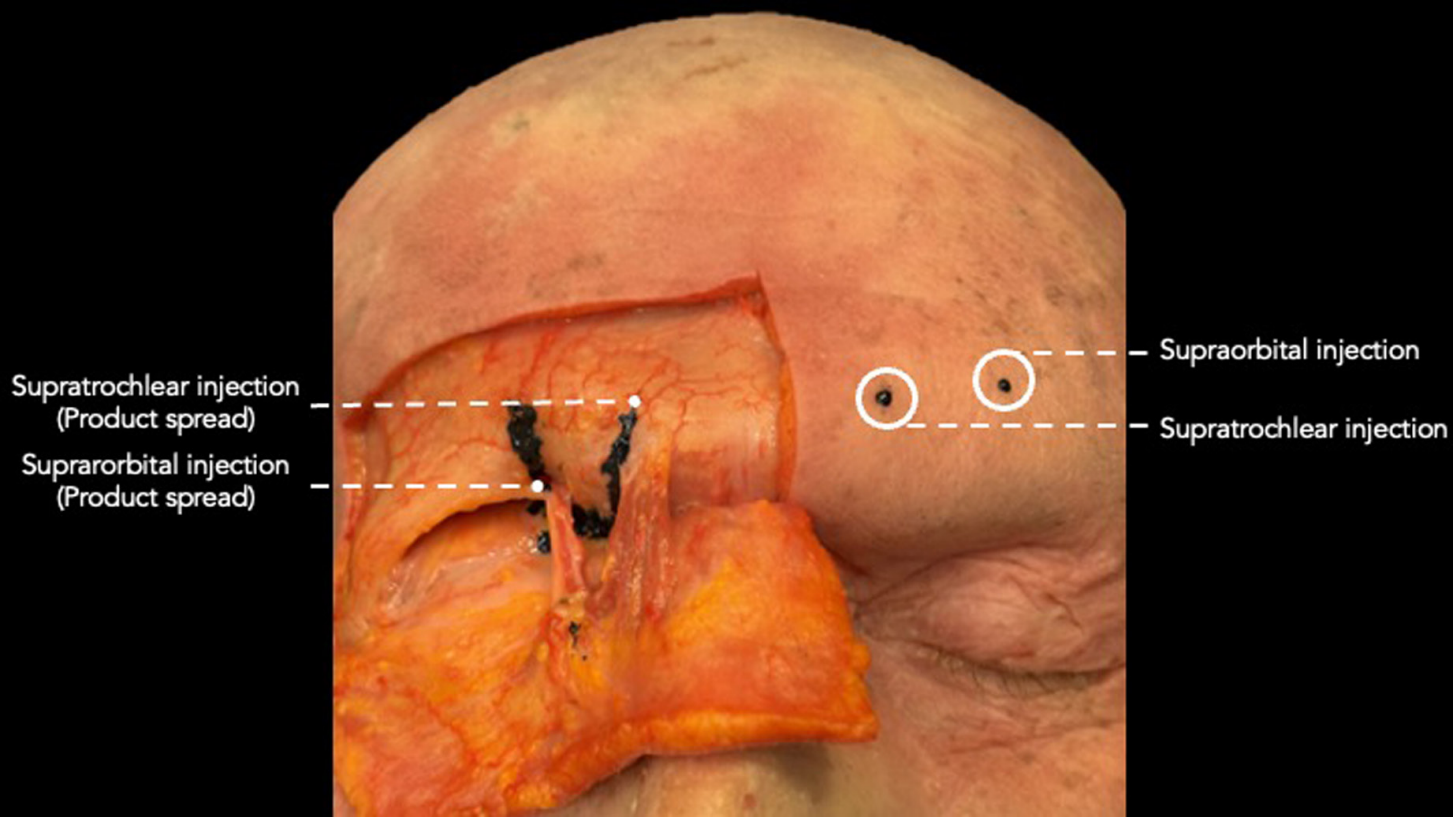
Dissection showing the supraorbital and supratrochlear injection location with their product spread upon deflection of the overlying soft tissues.

The supraorbital region was injected with a mixture of water and methylene blue coloring via a 31G BD syringe (BD, Franklin Lakes, NJ, USA) in two separate locations. The selected locations were:

– Supratrochlear region: Vertical line passing through the medial canthus in the middle of the hairy eyebrow, contact with bone.

– Supraorbital region: Vertical midpupillary line in the middle of the hairy eyebrow, contact with bone (Figures 1 and 2).

Both locations (supraorbital and supratrochlear) were injected into the supraperiosteal plane with constant bone contact during product administration with either 0.5 or 0.1 cc of colored material to mimic the spread of the product during neuromodulator injections. The material (water) was specifically selected to resemble the use of saline during clinical neuromodulator injections. The difference in molecular size between botulinum toxin type A and methylene blue was considered negligible and not a true influencing factor for the purposes of this study.

After the product was injected, the time until the periorbital soft tissues were reflected was approximately 5–10 min; this is the time it took to dissect and expose the injection site. No manual manipulation and no tissue movement were conducted, to identify the raw extent of product migration.

### 2.3 Anatomic Dissections

**FIGURE 3:**
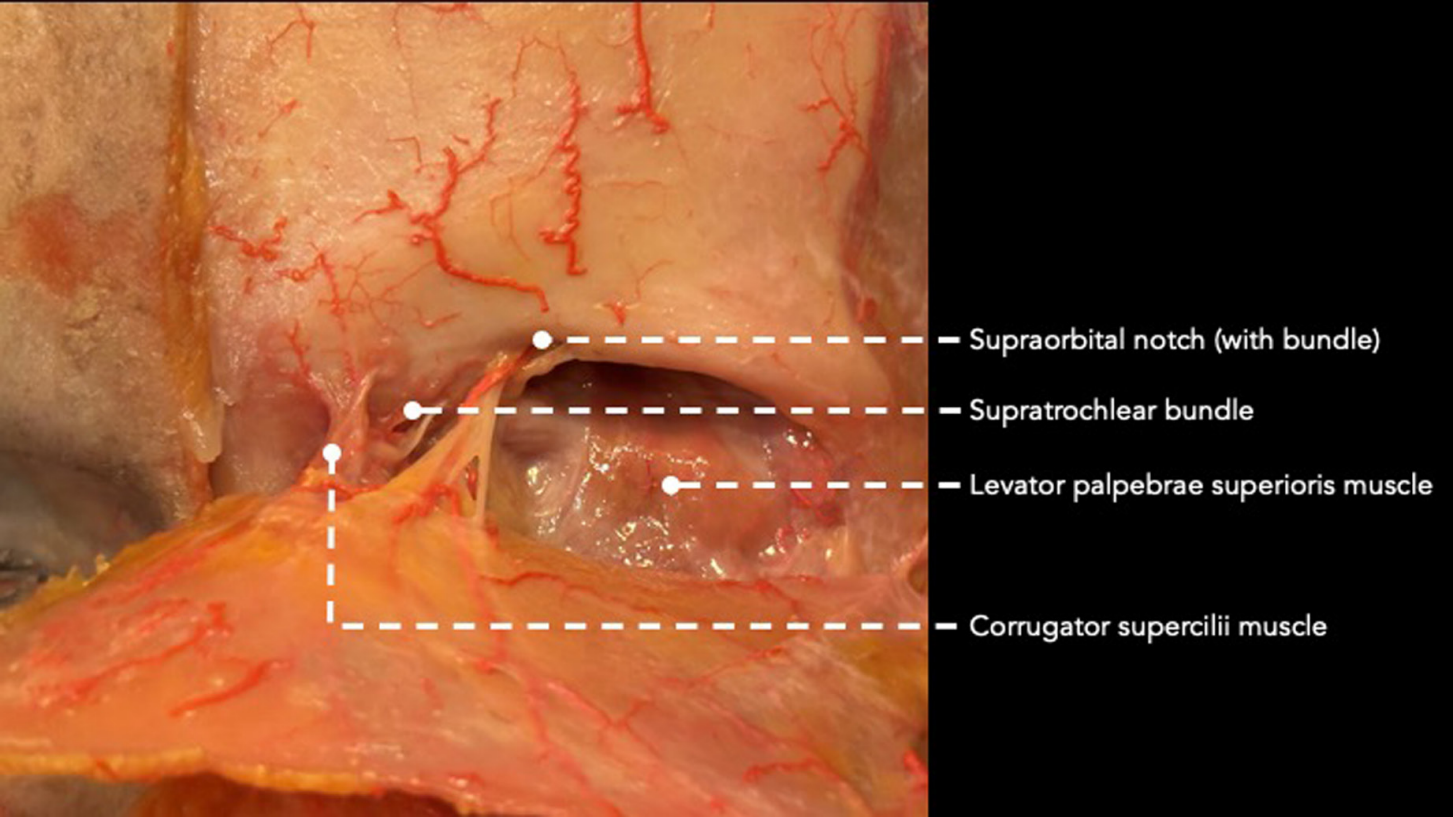
Dissection showing the supratrochlear and supraorbital neurovascular bundles, the CSM, and the LPS upon deflection of the overlying soft tissues.

After the injection procedure, the supraorbital region was dissected: A horizontal incision was made in each supraorbital region, starting 2 cm cranial to the bony superior orbital rim. The entire frontal soft tissue block was reflected caudally, exposing the orbital septum and the respective supratrochlear or supraorbital neurovascular bundle (Figure 3). Anatomic structures colored in blue product were considered to be affected by the injected material and were subsequently documented.

### 2.4 Statistical Analysis

Given the descriptive nature of this study, descriptive analyses were conducted using SPSS Statistics 23 (IBM, Armonk, NY, USA).

## 3 Results

### 3.1 General Description

Nine non‐embalmed human body donors (seven males and two females) were investigated in this study with a mean age at death of 72.8 (16.1) years (range: 43–83) of which seven were of Caucasian and two were of Chinese ethnic origin. The 18 supraorbital regions were injected 28 times (14 supratrochlear and 14 supraorbital) with 0.5 cc, whereas eight cases (four supratrochlear and four supraorbital) were injected with 0.1 cc of colored product.

### 3.2 Color Distribution: Supratrochlear

After injecting 0.5 cc in the deep supratrochlear region, only one injection procedure (out of 14 procedures; 7.1%) resulted in the coloring of the LPS. In all other cases (*n* = 13), the product stained the supratrochlear neurovascular bundle, the periosteum of the medial and superior orbit, the CSM, and the lower frontal soft tissues but without reaching the intra‐orbital space. In 11 cases (78.6%), the orbital septum was colored but did not reach the LPS, whereas in the single observed case where the product reached the LPS, the orbital septum was not colored in blue.

No color was detected inside the orbit or in close proximity to the LPS when 0.1 cc of material was injected.

### 3.3 Color Distribution: Supraorbital

**FIGURE 4:**
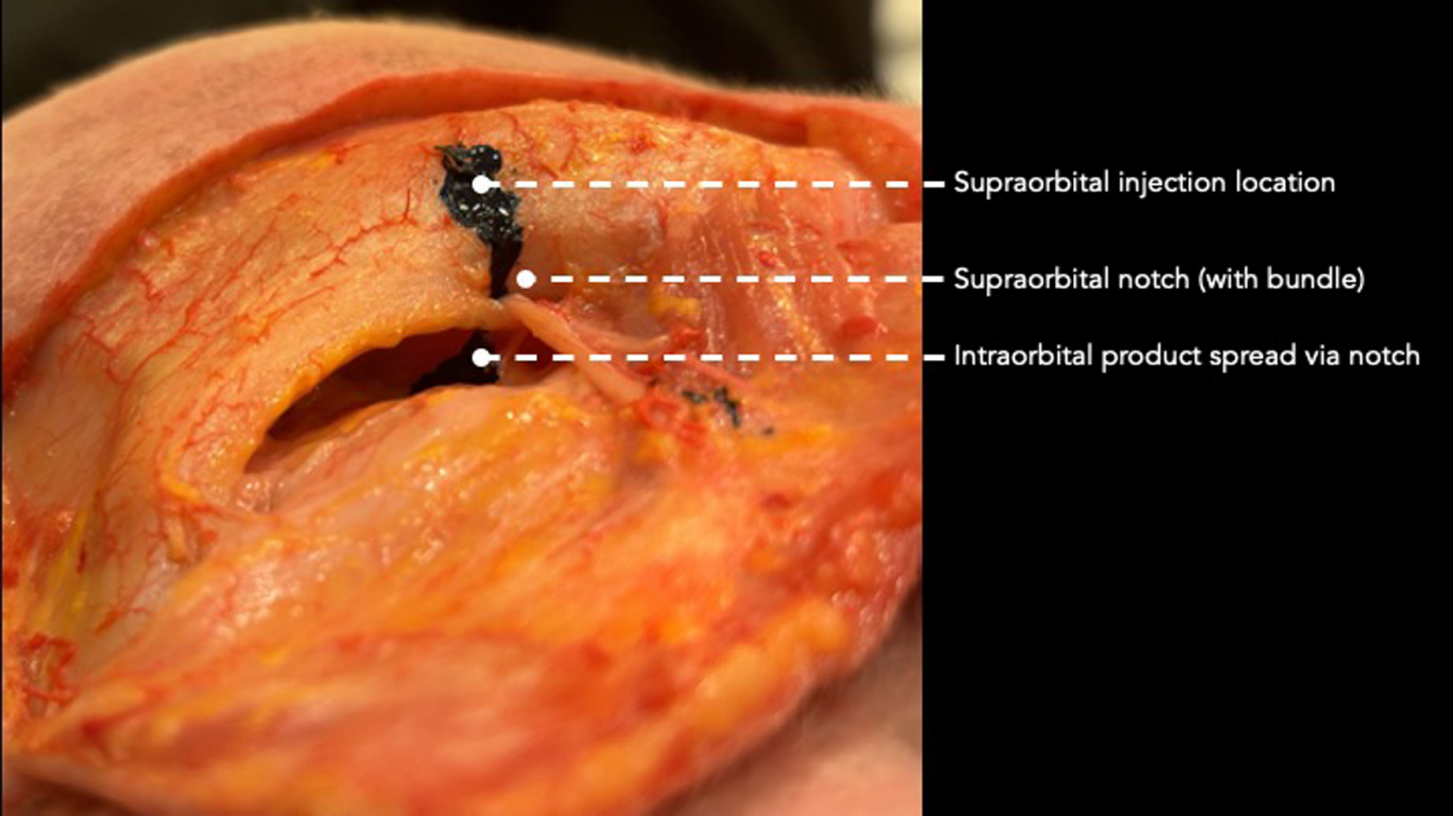
Dissection showing the intra‐orbital product spread after injection in the supraorbital region.

When injecting 0.5 cc into the deep plane of the central portion of the eyebrow in the location of the supraorbital foramen, six injection procedures (out of 14 procedures; 42.9%) resulted in the coloring of the intra‐orbitally located LPS and its respective Whitnall's ligament (Figure 4). In the other eight cases, the product stained the supraorbital neurovascular bundle, the periosteum of the central superior orbit, and the lower frontal soft tissue planes, however, it did not reach the intra‐orbital space. In nine cases (64.3%), the product stained the orbital septum; of those, five cases (35.7%) reached additionally the LPS, whereas in one case (7.1%), the LPS was stained by the product without staining the orbital septum.

No color was detected inside the orbit or in close proximity to the LPS when 0.1 cc of material was injected.

## 4 Discussion

In a previous publication on the topic of neuromodulator‐induced blepharoptosis, the authors identified that among anatomy and product‐related factors, the applied injection technique seemed to be the most influential factor for causing this adverse event type [12]. However, the authors did not provide a detailed anatomic description of the access pathway by which the product can travel from extra‐ to intra‐orbital to reach the LPS. The purpose of the present study was to identify such access pathways and to discuss how to reduce the risk of eyelid ptosis during neuromodulator injections.

The setup of our study was based on immobile human body donors, which lack tissue turgor pressure, pulsatile blood flow (venous and arterial), muscular activity, and which have a temperature of 4*°*C–10*°*C instead of 36*°*C–38*°*C. These study design limitations need to be accounted for when reviewing the results obtained. However, it should be assumed that all of the above tissue‐related factors can contribute to more product migration and diffusion in real (living) patients as they do not lack muscular activity, blood pressure, and normal body temperature. It is most likely that the mobility and the temperature of the tissue will allow the product to migrate more from the injection site into adjacent anatomic regions which in return should allow for the assumption that the deployed experimental model is under‐representing the true clinical reality.

The injection sites in this study are targeting the supratrochlear and the supraorbital neurovascular bundles because of the selected 2D (medial canthus and midpupillary) and 3D (bone contact) locations. This was specifically chosen to administer the product into danger areas to enable the product to reach the LPS. The results of the conducted anatomic dissections revealed that both locations carry the risk of contributing to eyelid ptosis. The supratrochlear injection site resulted in LPS staining in 2.78% (1 out of 36 injection procedures), whereas the supraorbital injection site resulted in 16.67% (6 out of 36 injection procedures) in LPS staining. However, it is important to note that only the 0.5 cc volume injections (*n* = 28) resulted in LPS staining, whereas no such event was observed when 0.1 cc of product were injected (*n* = 8). This difference in injected volume (between 0.5 and 0.1 cc) most likely indicates that the amount of injected volume plays a key role in causing upper eyelid ptosis. It is plausible that more injected fluid will have a greater likelihood to spread to adjacent anatomic regions than smaller amounts. A greater amount of fluid will increase the pressure of the surrounding tissues onto the administered material, squeezing the material into locations of lesser resistance. These locations represent passages by which the colored material can escape the pressure and by which the product can enter other anatomic compartments. These passages are the tissue tunnels formed by the supratrochlear and supraorbital neurovascular bundles. These bundles allow for the passage of the respective nerves, arteries, and veins to travel from intra‐orbital toward extra‐orbital and in return allowing the product to travel from extra‐ to intra‐orbital [13, 14]. The conducted dissections revealed that in 100% of the cases when the LPS was stained (*n* = 7), the respective neurovascular bundle was colored as well, most likely indicating that the bundle is the access pathway by which the product travels into the orbit.

In reverse, all injections conducted in this study (*n* = 36) stained the supratrochlear and supraorbital bundles but only in 2.78% (*n* = 1; supratrochlear) and in 16.67% (*n* = 6; supraorbital) it reached the LPS. This difference indicates that the supraorbital bundle is more prone to allow for the passage of product to reach the LPS than the supratrochlear bundle most likely due to the shorter distance to the LPS as compared to the supratrochlear bundle. It also indicates that despite the injection sites being specifically selected to reach the LPS, this event occurred only in a total of seven cases (19.44%) and only for the 0.5 cc volume injected.

It is interesting to note that the orbital septum was stained in a total of *n* = 28 injection procedures (77.78%) but only five of those cases (17.86%) displayed additionally a blue staining of the LPS. This aspect could potentially imply that the orbital septum has a certain degree of barrier function which seems to be stronger for the supratrochlear than it is for the supraorbital region.

Clinically, the results of this injection‐ and dissection‐based study suggest that there is a risk of causing upper eyelid ptosis when the deep regions of the supratrochlear and supraorbital locations are targeted, which is in line with previous publications and common clinical practice [15, 16]. A major influencing factor based on the results obtained in this study seems to be the injected volume. Despite the fact that in real clinical practice no one would inject 0.5 cc of product into the deep supraorbital region, it shows that all potential adverse events occurred at 0.5 and not at 0.1 cc injected volume. This would indicate that for glabella injections, a lower reconstitution volume could be selected (1.0 cc for a 100 I.U. vile) to reduce uncontrolled product spread into the intra‐orbital space. Depending on the type of neuromodulator and the reconstitution, a volume of 0.1 cc is common for the treatment of the CSM. However, it should be noted that in this study, no physical pressure was applied onto the injection site. When injecting neuromodulator in the region of the neurovascular bundle, bleeding often occurs after injuring one of the vessels. This is generally stopped by applying pressure, which could facilitate the spread of the neuromodulator product into the respective neurovascular bundle. Another possibility to increase safety and reduce the potential risk for upper eyelid ptosis is to not target the deep midpupillary supraorbital region but instead to inject strictly superficially. This can be achieved by angling the needle at a 10°–30° angle (when measured against the skin surface) and to have control of needle depth. The rationale for this injection technique is that the supraorbital neurovascular bundle is located deep, therefore the product application plane should be superficial. Creating a subdermal wheal or bump with the injected product might additionally help to remain in the superficial plane. When it comes to targeting the CSMs, a deep injection is recommended to best reach the muscle belly which originates at the supraciliary arch of the frontal bone [17, 18, 19]. This location is in close proximity to the supratrochlear foramen but angling the needle tip away from the orbit and pointing it toward the contralateral temporal region might be of advantage. Another way to best direct the needle is to align the end of the syringe (i.e., plunger plate) toward the ipsi‐lateral mandibular angle and advance it into the most medial lower corner of the hairy eyebrow until bone contact is established or the supraperiosteal plane is reached. The previously suggested recommendation to compress the bony orbital rim can be of support when trying to block the access pathway of the injected product via the supratrochlear and supraorbital foramina/notches. Removing the syringe but keeping the foramina/notches manually compressed for about 4–6 s after the injection is completed might increase the safety profile of the treatment (see Table 1).

**TABLE 1:** Safety recommendations based on the results of this study.

General	Supratrochlear location	Supraorbital location
Inject slowly with minimal plunger pressure	Point needle toward contralateral temple or angle from the ipsi‐lateral mandibular angle	Inject superficially not deep
Use low reconstitution volume for glabella injections		
Inject small amount of volume		
Compress supratrochlear/supraorbital foramen/notch during the injection procedure		
Keep supratrochlear/supraorbital foramen/notch compressed for 4–6 s after the injection procedure		

Although this study was conducted in human body donors and only *n* = 36 injection procedures were conducted, the results are in alignment with current clinical practice. The understanding of the layered arrangement of the face allows aesthetic injectors to select the plane of product application and by knowing in which plane the respective vessels or nerves travel, it enables them to play “hide and seek” with those structures: If the supraorbital neurovascular bundles are located deep, the product should be injected superficially [20]. When the location of the neurovascular bundle is known, the needle can be advanced away from the respective location understanding the difference between dermal penetration site and product application site. Future studies, however, will need to expand on the results presented herein by investigating a larger sample and conducting more targeted injection procedures.

## 5 Conclusion

The results of this injection‐ and dissection‐based study revealed that both the supratrochlear and the supraorbital neurovascular bundles are access pathways for injected neuromodulator products to reach the intra‐orbital space and affect the LPS muscle. Out of 36 conducted injection passes, seven (19.4%) resulted in affection of the sole elevator of the eyelid of which 100% occurred only at an injection volume of 0.5 and not at 0.1 cc. Clinically, the results indicate that a low injection volume, a superficial injection for the supraorbital location, and angling the needle tip away from the supratrochlear foramen (toward the contralateral temple) when targeting the CSMs, can increase the safety profile of an aesthetic neuromodulator treatment.

## Author Contributions

F.P., W.‐J.H., G.‐S.Z., J.B.G., M.M., and M.A. performed the research. F.P., W.‐J.H., M.M., M.A., S.B.H., and S.C. designed the research study. S.B.H., M.V.C., G.‐S.Z., and J.B.G. contributed essential reagents or tools. F.P., W.‐J.H., G.‐S.Z., M.A., S.C., and M.M. analysed the data. F.P., W.‐J.H., S.C., S.B.H., and M.A. wrote the paper.

## Conflicts of Interest

The authors declare no conflicts of interest.

## Data Availability

The study data are available from the corresponding author upon reasonable request.
